# Rejewski & Enigma

**DOI:** 10.1016/j.patter.2020.100011

**Published:** 2020-04-10

**Authors:** David Inman

**Affiliations:** 1 Science Museum, London, UK

## Abstract

Alan Turing and Bletchley Park are rightly recognized for their work on breaking the Enigma code. However, this was built on a foundation of work during the 1930s by the Polish cryptographer, Marian Rejewski. Often working alone, and with limited resources, he found ways to break early Enigma code. This article attempts to highlight the man and his invaluable contribution.

## Main Text

Enigma, made famous in the film *The Imitation Game*, was a code machine that the Germans used to send messages securely.^1^ It was regarded as impossible to crack by most of the German military, so critical information was sent. Yet the British, at Bletchley Park, found ways to break Enigma, and some speculate that this shortened the war by years.

Enigma is a cipher machine; that is, it substituted one letter with another to make an encrypted message that could be sent via radio. These messages were received and passed through another Enigma machine to show the original message. The radio traffic was picked up by the British, but decrypting the messages was so hard that the Germans had some confidence these communications were secure.

So why was Enigma so hard to crack? Some cipher machines before Enigma had fixed substitutions (e.g., A always became C, B always became D, and so on). These were easy to break because language uses some letters more than others (in English, E is rather common). You could look at the frequency of letters in the coded message and guess what the substitutions were. (Aside: Google Translate partially works like this.)

Enigma did much better than that. Every time you pressed a key on the keyboard, the settings (and hence the substitution) would change—if you pressed AAAA, you might get the code GQNL, for example. So frequency analysis didn’t help. But worse, there were so many settings. The settings were a combination of plug settings and rotor settings. Together, these made a total of 103,325,660,891,587,134,000,000 possible settings. If you checked one setting each minute, it would take much, much longer than the life of the universe to try them all. And the Germans changed the settings every day at midnight! No wonder the Germans were so confident it was secure.

So how was it cracked? Enigma had one fatal flaw, and a genius, hand-picked team at Bletchley Park exploited this flaw. The flaw was that the Enigma machine could never substitute one letter with the same letter (A could never be coded as A, for example). It was the way the machine was designed.

But how did this help? It needed a breakthrough insight. If you could guess part of the original message (“Heil Hitler,” perhaps), you could use that (called a “crib”) plus the Enigma machine flaw to dramatically reduce the possible settings to search for. By using electromechanical machines (Bombes), it was possible to find the settings in less than an hour and decode messages for that day before the settings changed again at midnight. There were many Bombes in Britain but also in the US. Transatlantic cables sent the cribs to the US, and possible settings were returned.

So who cracked Enigma? Alan Turing is often credited with this, for example in the film *The Imitation Game*, but many others at Bletchley Park such as Gordon Welchman were key. The work of the Polish cryptologists was also so important. They were decrypting German messages until 1940, when the Enigma procedures changed, and shared their knowledge with British codebreakers. A key person was Rejewski, often ignored, so I’d like to offer him some credit here.^2^

Breaking German codes was more urgent in Poland than the UK in the 1920s due to geography and the aftermath of WWI. Until 1923, Germany believed its secure codes were not broken and rejected Enigma as too expensive even if it offered extra security. However, the famous WWI Zimmerman Telegram was described in Churchill’s *The World Crisis* in 1923. This telegram had been assumed to be secure—an encrypted message offering Mexico parts of the US if they allied with Germany in WWI. It probably encouraged the US to enter WWI. After the German military realized its “secure codes” had been broken, it bought Enigma to enhance security.

The German military added extra features to Enigma, in particular the plug board to increase security. However, Rejewski, a Polish genius, found ways to read these Enigma messages and continued to do so until 1940, when extra security procedures were introduced. A major contribution was the invention of the Bombes, which speeded up the search for the Enigma settings for the day using guesses or cribs.

He provided essential experience to Bletchley at the start of WWII yet was sidelined after he escaped to Britain. The work at Bletchley was extensive by that time, yet he was only known to a few there. He wasn’t recognized until 30 years after the war. Secrecy was so tight at Bletchley—workers were never told how their info was used. For example, there was a dance at Bletchley the day before D Day. It wasn’t cancelled, as that might have aroused suspicion of an important event. In the end, D Day was delayed by 1 day due to weather. Bletchley workers had a chance to recover from the dance!^3^

So how important was breaking Enigma? Perhaps the Battle of the Atlantic gave the best evidence of how valuable breaking Enigma was. In 1942, the German navy changed its Enigma by adding an extra rotor. It took 9 months before this was broken and losses reduced. The figures of Allied shipping lost to U boats show the effect: 1940, 225; 1941, 288; 1942, 452; 1943, 203.^4^ Some estimate that the war was reduced by over a year because of the information Enigma provided.

Rejewski was key to this success. It wasn’t until 2000, however, that he was posthumously awarded Poland's second-highest civilian decoration, the Grand Cross of the Order of Polonia Restituta.^5^ In 2014, the Institute of Electrical and Electronics Engineers (IEEE) honored Rejewski with its prestigious Milestone Award, for achievements that have changed the world.

**Figure:**
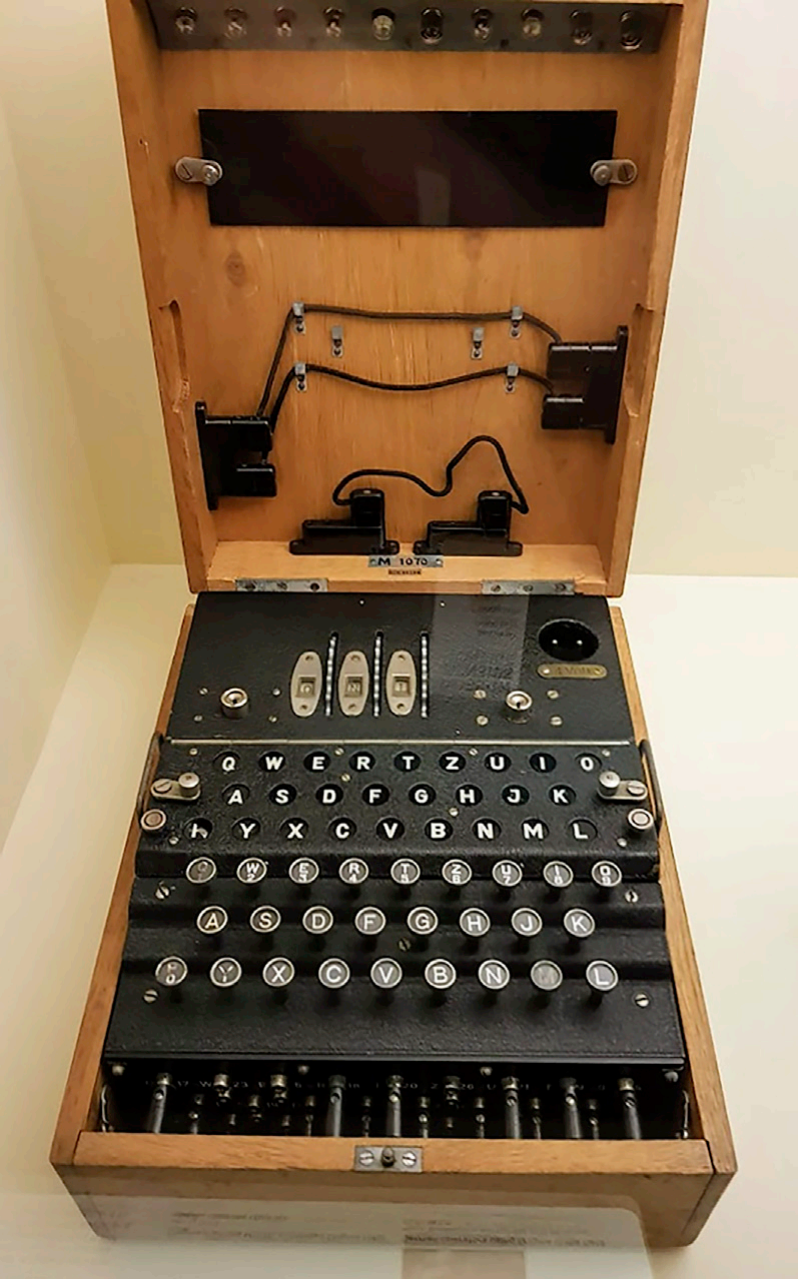
Image 1. Military Enigma Machine on Display at the Science Museum
